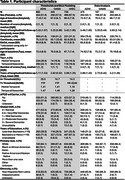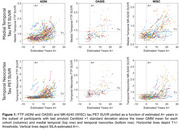# Factors influencing tau trajectories along the amyloid timeline from three cohorts

**DOI:** 10.1002/alz70856_098214

**Published:** 2025-12-24

**Authors:** Tobey J. Betthauser, Hailey Bruzzone, Jacob Morse, Finnuella Carey, Corinne D. Engelman, Richard J. Chappell, Carey E. Gleason, Lindsay R Clark, Brian A. Gordon, Megan L. Zuelsdorff, Rebecca E. Langhough

**Affiliations:** ^1^ University of Wisconsin‐Madison School of Medicine and Public Health, Madison, WI, USA; ^2^ University of Wisconsin School of Medicine and Public Health, Madison, WI, USA; ^3^ Washington University School of Medicine, Saint Louis, MO, USA

## Abstract

**Background:**

Recent studies demonstrate tau burden is heterogeneous after A+ onset and is temporally proximal to clinical impairment in sporadic AD. This study uses temporal modeling and neuroimaging data from three cohorts to investigate common factors that may hasten amyloid‐related tau accumulation.

**Methods:**

Participants with available amyloid and tau PET imaging were included from ADNI (*n* = 880), OASIS (*n* = 445), and University of Wisconsin (WISC: WRAP and Wisconsin ADRC; *n* = 739) cohorts. The following steps were completed separately for each cohort. Amyloid and tau were quantified, respectively, using Centiloids (CL) and medial temporal and temporal neocortex standard uptake value ratios (SUVR). A+ and T+ thresholds were defined as the mean plus two standard deviations (SDs) of lower Gaussian mixture model distributions. Sampled iterative linear approximation (SILA) was used to estimate A+ onset age (EAOA) and A+ time (age at observation minus EAOA). To understand moderators of the relationship between A+ time and tau SUVR's, we excluded those deemed confidently A‐ (<1 SD below the lower GMM group mean) and used LMEs to characterize associations between A+ time and tau SUVR, and investigated whether age at tau baseline, *APOE‐e4* carriage, sex, or education category explained additional variation in tau, both as main effects and interactions with A+ time.

**Results:**

Cohort characteristics are shown in Table 1. Results (Figure 1) were mostly consistent between OASIS and WISC cohorts with A+ time having a significant positive association with tau SUVR in both medial temporal and temporal neocortex, and *APOE‐e4* carriage having a significant interaction with A+ time for the medial temporal SUVR (*APOE‐e4* carriers had faster tau trajectories). These effects were also significant in ADNI, and additionally interactions of A+ time by baseline tau age and by sex (females had faster tau trajectories) were significant for medial temporal tau, and A+ time by baseline tau age for temporal neocortex (younger age had faster tau trajectories). The education by A+ time interaction did not reach significance in any cohort/region.

**Conclusion:**

In three longitudinal cohorts, *APOE‐e4* carriage consistently accelerated tau trajectories relative to A+ onset. Future work will further investigate these relationships and cohort differences that may contribute to mixed findings.